# Influence of Polymers on the Performance and Protective Effect of Cement-Based Coating Materials

**DOI:** 10.3390/ma18143321

**Published:** 2025-07-15

**Authors:** Yihao Yin, Yingjun Mei

**Affiliations:** School of Civil Engineering, Chongqing Jiaotong University, Chongqing 400074, China; yyhyddx@163.com

**Keywords:** cement-based coatings, polymers, interfacial bond strength, durability

## Abstract

Traditional cementitious coating materials struggle to meet the performance criteria for protective coatings in complex environments. This study developed a polymer-modified cement-based coating material with polymer, silica fume (SF), and quartz sand (QS) as the principal admixtures. It also investigated the influence of material composition on the coating’s mechanical properties, durability, interfacial bond characteristics with concrete, and the durability enhancement of coated concrete. The results demonstrated that compared with ordinary cementitious coating material (OCCM), the interfacial bonding performance between 3% Styrene Butadiene Rubber Powder (SBR) coating material and concrete was improved by 42%; the frost resistance and sulfate erosion resistance of concrete protected by 6% polyurethane (PU) coating material were improved by 31.5% and 69.6%. The inclusion of polymers reduces the mechanical properties. The re-addition of silica fume can lower the porosity while increasing durability and strength. The coating material, mixed with 12% SF and 6% PU, exhibits mechanical properties not lower than those of OCCM. Meanwhile, the interfacial bonding performance and durability of the coated concrete have been improved by 45% and 48%, respectively. The grey relational analysis indicated that the coating material with the best comprehensive performance is the one mixed with 12% SF + 6% PU, and the grey correlation degree is 0.84.

## 1. Introduction

Concrete structures are susceptible to salt corrosion and freezing and thawing [1,2], resulting in cracking or spalling, a decrease in structural serviceability, and even structural failure. The use of anti-corrosion coatings can effectively improve the durability of concrete. Traditional cement-based coating materials are difficult to meet the performance requirements of protective coatings in complex environments due to high brittleness, insufficient bonding performance, and poor corrosion resistance [3,4].

Compared to cementitious materials, polymer cementitious coating materials are suitable for concrete structural repair due to their fast curing properties, resistance to aggressive chemical and thermal conditions [5,6], good flexural and tensile properties, and high durability [7,8,9,10]. In recent years, polymers such as VAE powder and PU emulsion have demonstrated significant advantages in cement-based material modification. Studies have shown that VAE can enhance the bonding properties of cementitious materials and can form both physical and chemical bonds with various substrates [11,12,13]. PU exhibits excellent chemical resistance, corrosion resistance, high energy absorption, elongation, and adhesion properties [14]. Styrene–butadiene-based polymers improve mechanical properties, cohesion, and strength by bridging microcracks and enhancing adhesion, thereby increasing the compressive strength, flexural strength, and freeze–thaw resistance of coating materials [15,16]. However, polymers tend to degrade when exposed to ultraviolet radiation or humid and hot environments over time [17,18], and the mechanical and chemical resistance properties of the coating materials still fail to meet the requirements for long-term protection under complex conditions [19,20,21].

Polymer cementitious coating materials are improved by organic–inorganic composite action. The organic component improves the coating material’s flexibility, corrosion resistance, and densification [22,23,24]; the inorganic component enhances the coating material’s compactness and durability [25]. As a result, organic–inorganic composite coatings have gained significant attention due to their synergistic advantages [26]. However, current research on inorganic components mainly focuses on nanomaterials (e.g., SiO_2_). Although it can optimize the microstructure, it faces problems such as high cost, poor dispersion, and a complicated construction process, which limit its engineering applications [27,28,29]. In contrast, quartz sand, as a low-cost inert filler, can improve wear resistance and volume stability of coatings through physical filling effects [30]. Meanwhile, it avoids the performance fluctuation caused by nanomaterial agglomeration, which is more suitable for large-scale engineering promotion. Silica fume, with its higher specific surface area and smaller average particle size [31,32], can reduce porosity and enhance the durability and strength of cement-based materials [33,34,35].

To this end, this study proposes the development of a novel polymer cement-based composite coating material using styrene–butadiene emulsion (SBE) and PU polymer emulsion, SBR and vinyl acetate–ethylene (VAE) polymer powder, SF, and QS. The effects of material composition on the mechanical properties of the coating, its interfacial bonding performance with concrete, and the durability of concrete after painting the coating were systematically investigated. The findings provide theoretical guidance and technical support for the design and engineering application of protective coatings for concrete in complex environments, and are of significant engineering relevance for extending the service life of infrastructure.

## 2. Materials and Methods

### 2.1. Raw Materials

1. Cement

Ordinary Portland Cement P·O42.5R was used in this study; specific parameters are provided in Table 1 and Table 2.

2. Quartz sand

Three grades of sand were selected: 20–40 mesh, 40–70 mesh, and 70–140 mesh. The basic parameters are provided in Table 3.

3. Polycarboxylate Ether (PCE) Superplasticizer

The PC-6220 PCE was used; specific parameters are provided in Table 4.

4. Defoamer

The organic silicon defoamer ALLPON P801 was used; specific parameters are provided in Table 5.

5. Polymers

**Figure 1:**
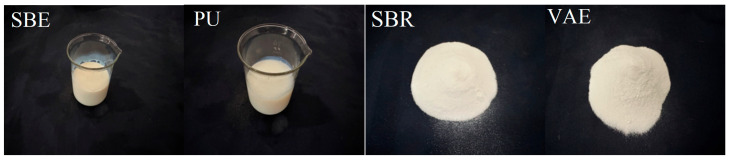
Polymers used in this study.

Two types of polymer emulsions and two types of polymer powders were selected to meet different working conditions requirements. The basic forms of the polymers are shown in Figure 1, and their technical specifications are provided in Table 6.

6. Silica fume

The specific parameters of silica fume are provided in Table 7.

### 2.2. Composition and Preparation of Coating Materials

#### 2.2.1. Design of the Mix Proportion for Polymer Cement-Based Coating Materials

1. Quartz Sand: A blend of 20–40 mesh, 40–70 mesh, and 70–140 mesh quartz sand was used, with the optimal ratio being 1:0.51:0.32. The blended quartz sand reached its densest state with a density of 1384 kg/m^3^.

2. Water-to-binder ratio (W/B): 0.34.

3. Binder-to-sand ratio: 1.5.

4. Polycarboxylate-based superplasticizer (PCE): The dosage of the water-reducing agent is adjusted based on workability.

5. Polymer: Percentage of the mass of the binder material, with dosages of 3%, 6%, and 9%.

6. SF: Replacing cement, with replacement rates of 8%, 12%, and 16%.

#### 2.2.2. Design of the Mix Proportion for OCCM

The mixing ratio design of OCCM follows points 1–4 of the mix proportion design for polymer cement-based coating materials in Section 2.2.1.

#### 2.2.3. Preparation of Coating Materials

1. Accurately weigh water, cement, quartz sand, polymer powder/emulsion, water-reducing agent, defoamer, and silica fume, and mix them according to the predetermined proportion.

2. Before stirring, moisten the inner wall and blades of the mixer, and use a moisture-absorbing towel to remove any excess water from the surface.

3. Next, add the materials in the order of water, quartz sand, cementing materials, polymer, water-reducing agent, and defoamer. To ensure the uniformity of the mixture, first stir slowly for 60 s, then wait for 60 s, and finally stir quickly for another 60 s.

4. For the preparation of OCCM, add water, cement, quartz sand, and water-reducing agent. The remaining steps are carried out according to the preparation method for polymer cement-based coating materials.

### 2.3. Test Methods

#### 2.3.1. Workability Test

In compliance with GB/T 2419-2005 [36]. the fluidity of the polymer cement-based coating was tested. In compliance with SL/T 352-2020 [37] the consistency of the coating was measured using a mortar consistency tester manufactured by Hebei Lucheng Instruments Company, Hengshui, China.

#### 2.3.2. Mechanical Performance Test

The strength of the coating material was tested according to GB/T 17671-1999 [38]. The specimen size was 40 mm × 40 mm × 160 mm.

#### 2.3.3. Bonding Performance Test

1. Interfacial bonding flexural strength test

The interfacial bonding flexural strength was tested by T/GDHS 011-2024 [39]. The specific procedure is as follows:

In the concrete raw materials, the coarse aggregate is limestone crushed stone with a grain size of 5–20 mm; the fine aggregate is medium sand with a fineness modulus of 2.66. The water–cement ratio is 0.40; the sand rate is 38%; and the water reducing agent dosage is 1%. The concrete specimens used in this paper are adopted from this proportion. The size of the molded beam specimen is 100 mm × 100 mm × 400 mm, and standard curing is for 28 d.

The beam specimens were cut into smaller beams of 100 mm × 100 mm × 160 mm, and the freshly cut surfaces were roughened.

The cut concrete blocks were placed into molds measuring 100 mm × 100 mm × 400 mm, with two old concrete blocks positioned at both ends and their cut surfaces facing inward.

After painting the coating material, new concrete was poured immediately. The composite beam specimens were then standardly cured for 3 d, 7 d, and 28 d, respectively.

A three-point bending test was used to evaluate the interfacial bond flexural strength. The loading rate was 0.02–0.05 MPa/s. The ultimate failure load was recorded, and the interfacial bond flexural strength was calculated according to Equation (1).

(1) $$f_{f}=\frac{FL}{bh^{2}}$$

where $f_{f}$ is the interfacial bonding flexural strength (MPa); F represents the maximum load (N); L represents the distance between supports, 300 mm; b is the specimen width, 100 mm; h is the specimen height, 100 mm.

2. Interfacial bond splitting tensile strength test

The formed size of the concrete specimen was 150 mm × 150 mm × 150 mm, and the standard curing was for 28 d. Each specimen was vertically cut in half, and the cut surfaces were roughened. After painting the coating material on the cut surfaces, the two halves were bonded together. Standard curing was 7 d and 28 d, respectively. The interfacial bond splitting tensile strength test was carried out by GB/T 50081-2019 [40] with a loading rate of 0.10 MPa/s. The test results were calculated using Equation (2).

(2) $$f_{ts}=\frac{2F}{\pi A}=0.637\frac{F}{A}$$

where $f_{ts}$ is the interfacial bond splitting tensile strength (MPa); F represents the maximum load (N); A is the area of the specimen’s splitting surface (mm^2^).

3. Interfacial pull-off bond strength test

The interfacial pull-off bond strength test was conducted by DL/T 5193-2021 [41]. The specific procedure is as follows:

The molding size is a 100 mm × 100 mm × 400 mm rectangular beam specimen, and standard curing is 28 d. Each beam was then cut into two specimens measuring 50 mm × 100 mm × 400 mm. One of the specimens was placed back into the original mold with the cut surface facing upward.

After painting coating materials with thicknesses of 1 mm, 3 mm, and 5 mm, new concrete was cast. The molded 100 mm × 100 mm × 400 mm composite beam specimens were standardly cured for 28 d, 56 d, and 90 d, respectively.

An angle grinder was used to polish the surface of the specimen. After grinding, holes were drilled, and the surface was cleaned. A strong epoxy AB adhesive was used to bond a 50 mm diameter pull-off head to the core column formed by the drilled hole.

A pull-off tester was used to extract the core column. The maximum pull-off force was recorded, and the interfacial pull-off bond strength between the coating material and the concrete was calculated using Equation (3).

(3) $$R=\frac{X}{S}$$

where X represents the maximum pull-off force (N); R  is the interfacial pull-off bond strength (MPa); S is the tensile area of the specimen (mm^2^).

#### 2.3.4. Durability Performance Test

1. Accelerated freeze–thaw test

A series of freeze–thaw durability tests were performed following the specification DL/T 5150-2017 [42]. The quality loss and strength loss were measured at 50, 100, 150, and 200 cycles. Before the test, all specimens were soaked in water for 4 days. The temperature range of (−18 ± 2) °C to (5 ± 2) °C was considered in the freeze–thaw cycles and one complete cycle for 3.5 ± 0.5 hrs. The coating specimens have dimensions of 40 mm × 40 mm × 160 mm, and the coated concrete specimens have dimensions of 100 mm × 100 mm × 100 mm with a coating thickness of 2 mm. The mass and strength loss rates are calculated using Formulas (4) and (5).

(4) $$\Delta W_{n}=\frac{W_{0}-W_{n}}{W_{0}}\times 100\%$$

(5) $$\Delta f_{n}=\frac{f_{0}-f_{n}}{f_{n}}\times 100\%$$

where $\Delta W_{n}$ and $\Delta f_{n}$ represent the specimens’ mass and strength loss rate after the nth cycle, respectively (%); $W_{0}$ and $f_{0}$, $W_{n}$ and $f_{n}$ are the masses and strengths of specimens before and after the experiments, respectively (MPa).

2. Sulfate corrosion resistance test

The sulfate resistance test was conducted on the coating material and the concrete protected with the coating material by GB/T 50082-2009 [43].

The size of the test specimen of coating material is 40 mm × 40 mm × 160 mm, and the size of the concrete specimen protected by coating material is 100 mm × 100 mm × 100 mm. The thickness of the coating is 1 mm, 2 mm, and 3 mm, respectively.

After standard curing for 28 days, the specimens were moved to a drying oven set at (80 ± 5) °C. After drying, the specimens were allowed to cool naturally. The specimens were then immersed in a 5% sodium sulfate solution for 7, 28, and 90 days, respectively. The compressive strength of the specimens was tested after immersion. The compressive strength loss rate was calculated using Equation (6).

(6) $$K_{f_{c}}=\frac{f_{c_{2}}-f_{c_{0}}}{f_{c_{0}}}$$

where $K_{f_{c}}$ is the strength loss rate (%); $f_{c_{0}}$ is the compressive strength without soaking (Mpa); $f_{c_{2}}$ is the compressive strength after soaking (MPa).

## 3. Results and Discussion

### 3.1. Coating Material Performance Testing and Analysis

#### 3.1.1. Workability

The target consistency is controlled within (95 ± 5 mm) by adjusting the PCE content. The specific workability is shown in Table 8.

It can be seen from the data in Table 8, the incorporation of VAE improves the workability of the coating material; the higher the dosage, the better the workability. With the addition of SBE, the required amount of water-reducing agent gradually decreases, indicating that SBE significantly improves the coating material’s flowability. In contrast, the incorporation of SBR and PU hurts the workability of the cement-based coating, requiring additional water-reducing agents to adjust its workability.

The workability of the polymer cement-based coating material deteriorates after the addition of SF and defoamer, and more water-reducing agent is needed to improve its workability.

#### 3.1.2. Mechanical Properties

After standard curing for 28 days, coating materials incorporating polymer emulsion or polymer powder were tested for compressive and flexural strength. The test results are shown in Table 9.

It can be seen from the data in Table 9 that the incorporation of polymer reduced the 28-day compressive strength of the cement-based coating materials.

Incorporation of no more than 6% VAE enhances the flexural strength of the coating material, with the highest 28 d flexural strength reaching 10.2 MPa, which is 10.8% higher than that of OCCM.

The addition of SF and defoamer significantly improved the compressive strength of the polymer cement-based coating material. The highest compressive strength of the coating material was achieved with 6% VAE + 16% SF, with the highest compressive strength of 65.1 MPa; the greatest increase in compressive strength was achieved with 3% SBE + 8% SF, with the compressive strength increasing by 88.1% compared with that of the coating material without SF.

The effects of the polymers selected in this paper and existing commercial polymers on the compressive strength and flexural strength of coating materials are summarized in Table 10. Negative numbers in the table indicate a decrease in strength, while positive numbers indicate an increase in strength.

Based on the results of workability and mechanical property tests, the optimal addition amounts for VAE, SBR, PU, and SBE in the coating material are 6%, 3%, 6%, and 3%, respectively. The corresponding optimal amounts for silica fume are 8%, 8%, 12%, and 8%, and the defoamer dosage is 0.1% in all cases.

#### 3.1.3. Freeze–Thaw Resistance

**Figure 2:**
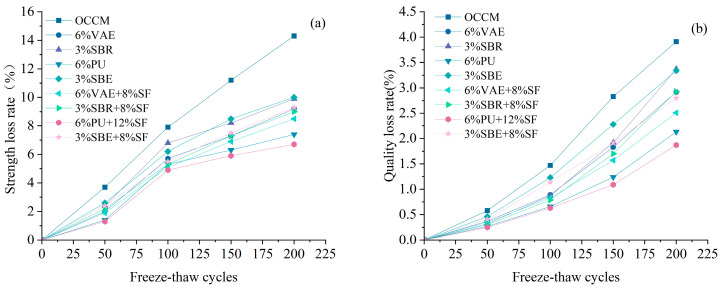
Strength loss rate (**a**), quality loss rate (**b**).

The molded coating material specimens were subjected to a rapid freeze–thaw test after standard curing for 28 d. The test obtained the rate of quality loss and the rate of strength loss of the coating material, as shown in Figure 2.

According to Figure 2, the coating material with 6% PU + 12% SF exhibited the best freeze–thaw resistance, with the mass loss rate and strength loss rate reduced by 52.1% and 53.1%, respectively, compared to OCCM.

The quality loss rate and strength loss rate of different types of polymer cement-based coating materials were lower than those of OCCM. The slope of the curves was flatter, and the higher the number of freeze–thaw cycles, the larger the reduction. The compounding of silica fume can significantly improve the freeze–thaw resistance of polymer cement-based coating materials.

#### 3.1.4. Sulfate Corrosion Resistance

**Figure 3:**
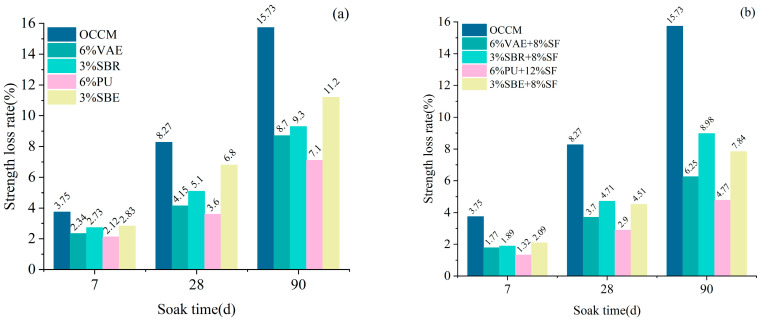
Strength loss rate: mono-doped polymer (**a**), doped with SF (**b**).

The molded coating material specimens were subjected to anti-sulfate erosion test after standard curing for 28 d, and the rate of loss of strength of the coating material was tested, as shown in Figure 3.

According to Figure 3, it can be seen that under the same polymer dosage and with a soak time of 90 days, the coating material with 3% SBR exhibited 16.9% higher resistance to sulfate attack compared to that with 3% SBE. Similarly, the coating material with 6% PU showed 18.3% higher sulfate resistance than that with 6% VAE.

When SF was incorporated into the polymer cement-based coating material, the rate of mechanical property degradation under sulfate attack was significantly reduced. In particular, the coating material with 6% PU + 12% SF exhibited the most pronounced improvement in sulfate resistance, which is 32.8% higher than that without silica fume, and 69.6% higher than that of OCCM.

### 3.2. Testing and Analysis of the Bonding Properties at the Interface Between Coating Materials and Concrete

#### 3.2.1. Interfacial Bonding Flexural Strength

**Figure 4:**
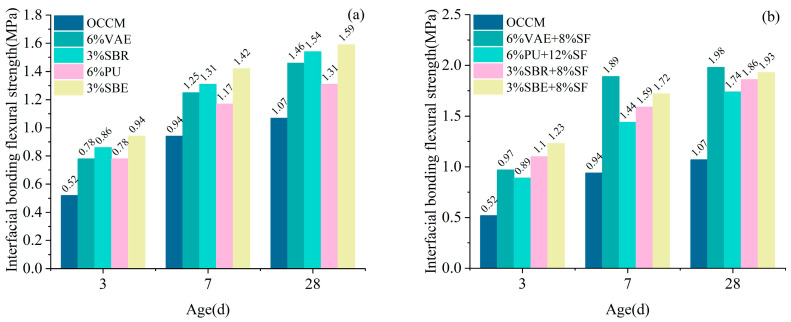
Interfacial bonding flexural strength: mono-doped polymer (**a**), doped with SF (**b**).

The molded specimens were subjected to interfacial bonding flexural strength tests after standard curing for 3 d, 7 d, and 28 d. The interfacial bonding flexural strength between the coating material and concrete was obtained from the test, as shown in Figure 4.

Different types of polymer cementitious coating materials can improve the interfacial bond flexural strength between coating materials and concrete. The coating materials with 6% VAE + 8% SF have the best effect in improving the interfacial bond flexural strength between coating materials and concrete at the age of 28 d, which is 85% higher than that of OCCM.

The addition of SF significantly improved the interfacial flexural bond strength between coating materials and concrete. The interfacial bond flexural strength of the coating material with concrete doped with 6% VAE + 8% SF increased by 24.4% at the age of 3 d compared with that of doped with 6% VAE alone; at the age of 28 d, this enhancement increased to 35.6%. The incorporation of SF enhanced the early-age bond strength and led to even more pronounced improvements at later ages, highlighting its important role in improving the bonding performance of cement-based coatings.

#### 3.2.2. Interfacial Bond Splitting Tensile Strength

**Figure 5:**
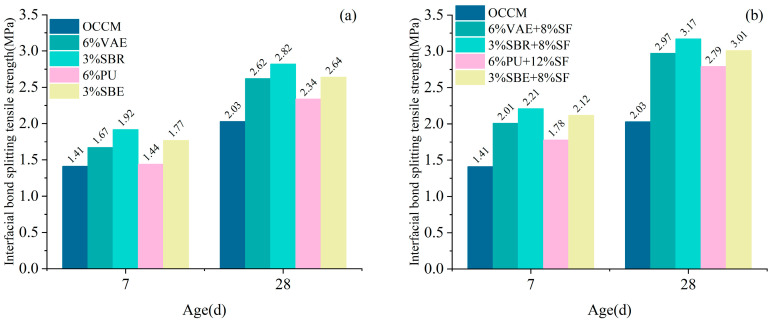
Interfacial bond splitting tensile strength: mono-doped polymer (**a**), doped with SF (**b**).

The molded specimens were subjected to interfacial splitting tensile tests after standard curing for 7 and 28 days. The interfacial splitting tensile strength between the coating material and concrete was obtained from the test, as shown in Figure 5.

It can be seen that different types of polymer cement-based coating materials can improve the interfacial bond splitting tensile strength between the coating materials and concrete. At the age of 28 d, the coating material with 3% SBR + 8% SF exhibited the highest interfacial bond splitting tensile strength, with a 56% increase compared to OCCM. This was followed by the 6% VAE + 8% SF coating, which showed a 46% improvement over OCCM.

The addition of SF significantly enhanced the early-age interfacial bond splitting tensile strength between the coating and concrete. Notably, the interfacial bond splitting tensile strength of 3% SBR + 8% SF coating material at 7 d age was also 8% higher than that of OCCM at 28 d age.

#### 3.2.3. Interfacial Pull-Off Bond Strength

The specimens after molding were subjected to interfacial pull-off bond strength test after standard curing for 28 d, 56 d, and 90 d, and the interfacial pull-off bond strength of the specimens was obtained from the test, as shown in Table 11.

According to the findings in Table 11, the coating material with 6% VAE + 8% SF had the strongest pull-off bond strength, showing a 43% improvement compared to OCCM and a 72% improvement compared to the uncoated control.

At the age of 90 d and the coating thickness of 5 mm, the 3% SBE + 8% SF coating material achieved the highest interfacial pull-off strength, with a 30% increase compared to OCCM. For the rest of the coating materials, the interfacial pull-off bond strength between the coating materials and the concrete showed a trend of increasing and then decreasing with the increase in the coating thickness at the age of 90 d. The maximum interfacial pull-off bond strength between the coating material and concrete was observed at a coating thickness of 3 mm.

#### 3.2.4. Microstructure and Water Absorption Analysis

**Figure 6:**
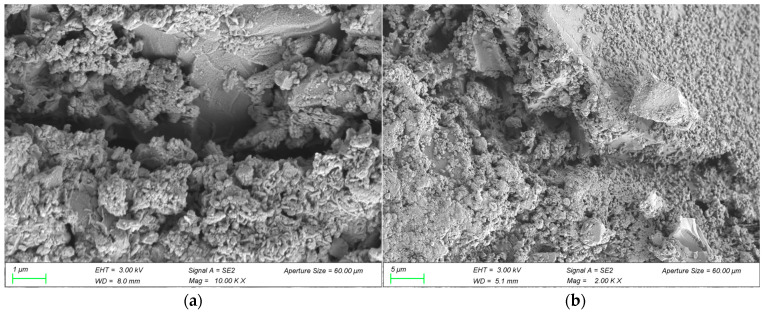
Microstructure of the bonded surface between OCCM and concrete (**a**), microstructure of the bonded surface between 6% VAE + 8% SF coating materials and concrete (**b**).

The above experiments analyzed the interfacial bonding performance between polymer cement-based coating materials and concrete from a macro perspective, but lacked a microscopic analysis of the interfacial bonding mechanism and an exploration of the pore structure of the coating materials. Therefore, SEM testing was used to obtain the microscopic structure of the bonding surface, as shown in Figure 6. Water absorption experiments were used to test the internal density of the coating materials, as shown in Table 12.

As can be seen from Figure 6, the 6% VAE + 8% SF coating material has a tighter bond with the concrete surface. The reason is that VAE forms a polymer film at the interface, further improving bonding performance and enhancing interface toughness [46]. SF particles are covered by Ca(OH)_2_ and fill the interface transition zone between the coating material and concrete, serving a physical micro-filling function. Reducing defects in the interface transition zone increases density, thereby improving the interface bonding performance between the coating material and concrete. The C-S-H gel formed by the reaction between SiO_2_ in silica fume and Ca(OH)_2_, which is a product of cement hydration, can strengthen the interface transition zone and improve bonding strength [47].

Table 12 shows that VAE forms a continuous film inside the coating material after curing. These films block capillaries and microcracks, preventing moisture from freely entering the coating material. At the same time, silica fume is a microfine particle that can fill the voids in concrete protective materials, reducing water penetration pathways. The reaction between silica fume and hydration products can generate more gel, making the coating material more compact.

### 3.3. Testing and Analysis of the Protective Effect of Coating Materials on Concrete

#### 3.3.1. Effect of Coating Materials on the Freeze–Thaw Resistance of Concrete

**Figure 7:**
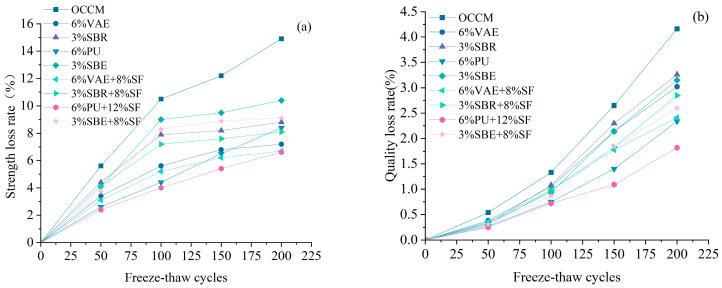
Strength loss rate (**a**), quality loss rate (**b**).

Different kinds of coating materials were painted onto concrete specimens with standard curing for 28 d. A freeze–thaw cycle test was carried out after 28 d of standard curing to obtain the quality loss rate and strength loss rate of concrete after protection with different kinds of coating materials, as shown in Figure 7.

Figure 7 shows that during the first 100 freeze–thaw cycles, the mass loss rate of concrete specimens coated by various types of polymer-cement-based coatings is similar, and the slope is reasonably flat. After 100 cycles, the slope of the curve for concrete specimens protected by OCCM begins to gradually increase, and the quality loss rate stays higher than that of the control group, which is protected by polymer-cement-based coatings.

Comparing the performance of the strength loss rate and mass loss rate of concrete specimens protected by four polymer-cement-based coatings incorporating SF after freeze–thaw cycles, the coating material with 6% PU + 12% SF shows the best performance. After 200 freeze–thaw cycles, the quality loss rate and strength loss rate are reduced by 56% and 55%, respectively, compared to concrete specimens protected by OCCM.

#### 3.3.2. Effect of Coating Materials on the Sulfate Corrosion Resistance of Concrete

Different types of coating materials were painted on concrete specimens that had undergone standard curing for 28 d. After 28 days of standard curing, the sulfate corrosion resistance test was performed. And the strength loss rates of the concrete specimens protected by different types of coating materials were obtained, as shown in Table 13.

The statistics in Table 13 show that the coating material with 6% PU + 12% SF gives the greatest protection. The sulfate corrosion resistance of concrete protected by this coating material is improved by 41% compared to concrete protected by OCCM, and by 61% compared to concrete without any coating material.

The greater the thickness of the coating material painted, the lower the strength loss rate of the concrete when the immersion time was 90 d. The concrete protected by 6% PU + 12% SF coating material has a 19% increase in sulfate erosion resistance at a coating thickness of 3 mm compared with 2 mm, and the coating thickness is recommended to be 3 mm. The rest of the coating material has a limited increase in sulfate erosion resistance at a coating thickness of 3 mm compared with 2 mm, and the coating thickness is recommended to be 2 mm for comprehensive economic considerations.

## 4. Gray Correlation Analysis Between Coating Performance and Types

Gray correlation analysis is an important part of gray system theory, developed by Deng in 1982 [48]. Generally, black is represented as a lack of information, and white is full of information. Thus, the information that is either incomplete or undetermined is called Gray. Gray correlation analysis can be used to analyze an unknown system with limited data. This method has been successfully used in exploring the main factors affecting the various behaviors of cement concrete [49,50,51].

The principle of gray correlation analysis is shown below.

$1.\;X_{i}$ is the factor behavioral sequence, and Y  is the system characteristic behavioral sequence.

(7) $$Y=\left\{Y(k),k=1,2,\ldots ,m\right\}$$

(8) $$X_{i}=\left\{X_{i}(k),k=1,2,\ldots ,m\right\},(i=1,2,\ldots ,n)$$

2. Dimensionless processing of variable data; mean normalization equation is as follows:

(9) $$X_{i}(k)=\left\{\frac{X_{i}\left(k\right)}{X_{1}},k=1,2,\ldots ,m\right\},(i=1,2,\ldots ,n)$$

3. Calculating the difference sequence:

(10) $$\Delta_{i}(k)=\left|Y(k)-X_{i}\left(k\right)\right|,(k=1,2,\ldots ,m)$$

4. Calculating the maximum difference and minimum difference between the two poles:

(11) $$M=\underset{i}{max}\underset{k}{max}\Delta_{i}(k)\;,\;N=\underset{i}{min}\underset{k}{min}\Delta_{i}(k)$$

5. Calculating the correlation coefficients:

(12) $$\varepsilon_{i}(k)=\frac{N+\xi M}{\Delta_{i}(k)+\xi M}\;,\xi \in (0,1)$$

6. Calculating the degree of gray correlation:

(13) $$r_{i}=\frac{1}{m}\sum_{k=1}^{m}\varepsilon_{i}(k),(k=1,2,\ldots ,m)$$

In this study, mechanical properties, bonding properties, durability properties, and comprehensive properties are defined as the system characteristic sequences. Polymer-modified cement-based coatings with different formulations are considered as the factor sequences. The closer the correlation is to 1, the better the performance of the coating material.

**Figure 8:**
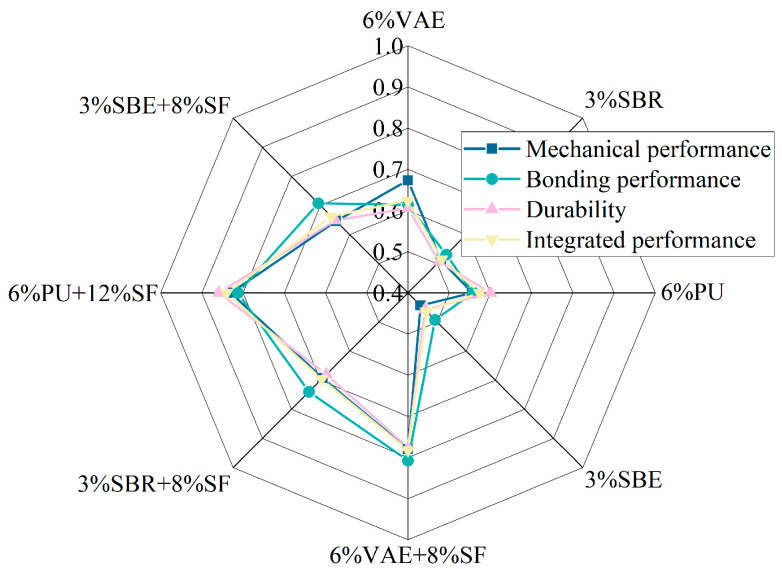
Gray correlation.

Figure 8 is a radar chart that shows the gray relational degree of polymer-cement-based coating materials with mechanical performance, bonding performance, durability, and overall performance for all mix ratios. For example, when the application scenario requires higher mechanical performance of the polymer-cement-based coating, the weighting coefficient for mechanical performance is set to 2, and the weighting coefficients for other properties are set to 1. The 6% PU + 12% SF coating material has the highest correlation with mechanical performance, with a gray relational degree of 0.83. This is followed by the 6% VAE + 8% SF coating material, with a gray relational degree of 0.78. When the application scenario requires higher bonding performance of the polymer-cement-based coating, the weighting coefficient for bonding performance is set to 2, and the weighting coefficients for other properties are set to 1. The 6% VAE + 8% SF coating material has the highest correlation with bonding performance, with a gray relational degree of 0.81, followed by the 6% PU + 12% SF coating material, with a gray relational degree of 0.80. The results indicate that the incorporation of SF significantly improves the mechanical, bonding, and durability properties of the polymer-cement-based coatings, and the best overall performance is exhibited by the 6% PU + 12% SF coating material.

## 5. Conclusions and Future Research

This study investigated the influence of material composition on the workability, mechanical properties, and durability of coating materials. The interface bonding performance of the coating materials with concrete was studied by evaluating the interfacial bond splitting tensile strength, the interfacial bond splitting tensile strength, and the interfacial pull-off bond strength. Additionally, the effect of protective coating materials on the durability of concrete was explored. The results indicate that the following:

1. The addition of SBR and PU will lead to poor workability of the coating materials, while the incorporation of VAE and SBE improves the workability of the coating materials. When active additives such as SF and defoamer are incorporated into the polymer-cement-based coating materials, the workability of the cement-based coatings deteriorates. However, SF, with its higher specific surface area and smaller average particle size, can enhance the strength of the coating material. Based on the balance between the workability and mechanical properties of the coating materials, the optimal incorporation levels of VAE, SBR, PU, and SBE are 6%, 3%, 6%, and 3%, respectively. The corresponding optimal amounts for SF are 8%, 8%, 12%, and 8%, and the defoamer dosage is 0.1% in all cases.
2. Different types of polymer-cement-based coating materials can improve the interface bonding strength between the coating material and concrete. The 6% VAE + 8% SF coating material shows the best improvement in interface bonding strength with concrete. At the age of 28 d, the interface bond flexural strength, interface bond splitting tensile strength, and interface pull-off bond strength are increased by 85%, 46%, and 43%, respectively, compared to OCCM. Microstructure and water absorption analyses indicate that the film-forming effect of polymers and the microfilling effect of silica fume can significantly improve the adhesion and mechanical properties of coating materials. The application cost of the 6% VAE + 8% SF coating material is 2.28 CNY/L. Considering both the economic cost and bonding strength factors, the optimal coating thickness is recommended to be 3 mm.
3. Concrete protected by polymer-cement-based coating materials performs better than concrete protected by OCCM in resisting freeze–thaw cycles and sulfate corrosion. The 6% PU + 12% SF coating material performs the best. After 200 freeze–thaw cycles, the quality loss rate and strength loss rate are reduced by 56% and 55%, respectively, compared to concrete protected by OCCM. After soaking for 90 days, the sulfate corrosion resistance is improved by 41% compared to concrete protected by OCCM, and by 61% compared to concrete without any coating. The application cost of the 6% PU + 12% SF coating material is 3.23 CNY/L. Considering both the economic cost and bonding strength factors, the recommended coating thickness is 3 mm, while the recommended coating thickness for the other materials is 2 mm.
4. The incorporation of SF significantly improves the mechanical, bonding, and durability properties of polymer-cement-based coating materials. The best overall performance is the coating material with 6% PU + 12% SF, with a gray relational degree of 0.84. This is followed by the 6% VAE + 8% SF coating material, with a gray relational degree of 0.78.

This study only considered the impact of a single factor on durability. Subsequent studies will consider the combined effects of multiple factors on coated materials. For example, how they exacerbate physical damage to coated materials and accelerate chemical corrosion. At the same time, the synergistic effects of these combined factors may form a self-accelerating vicious cycle.

## Figures and Tables

**Table 1:** Parameters of cement.

Physical Examination		Standard Value	Test Value
Solidification time	Condensation (min)	≥45	200
	Congeal (min)	≤600	263
**Chemical composition**		**Standard value**	**Test value (%)**
MgO		≤5.0	2.85
SO_3_		≤3.5	3
LOSS		≤5.0	3.4
Cl^−^		≤0.06	0.022
Al_2_O_3_		≤5	4.04
Fe_2_O_3_		≤5	4.03
CaO		/	53.4
SiO_2_		/	22.1
**Type of mixed material**		**Test value (%)**	
Slag		11	
Pozzolanic Material		1.6	
Limestone		3	
Natural Gypsum		6	

**Table 2:** Mechanical properties of cement.

	Age (d)	Standard Value	Measured Value
Compressive strength (MPa)	3	≥17.0	25.2
	28	≥42.5	48.3
Flexural strength (MPa)	3	≥3.5	5.0
	28	≥6.5	7.1

**Table 3:** Quartz sand specifications.

Mesh Size	Packing Density (kg/m^3^)	Apparent Density (kg/m^3^)	Porosity (%)
20~40	1146.2	1550	57.4
40~70	1203.7	1620	55.2
70~140	1253.7	1760	53.4

**Table 4:** Polycarboxylate-based superplasticizer technical parameters.

	Standard
Packing density (kg/m^3^)	400–700
Moisture content (%)	5
PH	7–9
Chloride content (%)	0.05
Concrete Water Reduction Rate (%)	25

**Table 5:** Defoamer parameters.

Active component (%)	~65
Appearances	White powder
Density (20 °C)	350 g/L
Solubility in water	Dispersible in water
PH	7

**Table 6:** Polymer specifications.

Types	Solid (%)	PH	Stickiness (MPa/s)	Film-Forming Temperature (°C)	Density (g/cm^3^)
SBE	50	8	120	−12	1.01
SBR	99 ± 1	3–7	\	7	0.5
PU	35	6–7	800–1500	1	1.2
VAE	98	6–9	\	0 ± 2	0.45

**Table 7:** Silica fume specifications.

SiO_2_ (%)	K_2_O (%)	Specific Surface Area (m^2^/kg)	Loss on Ignition (%)	Activity Index (%)
95.25	0.32	20,526	3.2	125

**Table 8:** Coating workability.

Coating Materials	PCE (%)	Flowability (mm)	Consistency (mm)
OCCM	0.11	210	95
3% VAE	0.16	200	96
6% VAE	0.12	206	99
9% VAE	0.03	220	102
3% SBR	0.15	200	95
6% SBR	0.16	210	105
9% SBR	0.18	180	100
3% PU	0.20	190	95
6% PU	0.30	185	92
9% PU	0.29	190	98
3% SBE	0.11	214	105
6% SBE	0.08	200	95
9% SBE	0.04	208	110
6% VAE + 8% SF	0.11	201	91
6% VAE + 12% SF	0.14	200	92
6% VAE + 16% SF	0.18	210	96
3% SBR + 8% SF	0.24	190	93
3% SBR + 12% SF	0.27	185	95
3% SBR + 16% SF	0.30	185	91
6% PU + 8% SF	0.24	190	91
6% PU + 12% SF	0.40	180	95
6% PU + 16% SF	0.44	185	92
3% SBE + 8% SF	0.17	210	92
3% SBE + 12% SF	0.21	210	90
3% SBE + 16% SF	0.26	230	98

**Table 9:** Mechanical properties of coatings.

Coating Materials	Compressive Strength (MPa)	Flexural Strength (MPa)
OCCM	56.8	9.2
3% VAE	45.8	7.6
6% VAE	52.8	10.2
9% VAE	52.9	9.6
3% SBR	43.2	7.0
6% SBR	47.2	6.7
9% SBR	51.3	8.4
3% PU	33.9	5.9
6% PU	36.9	7.6
9% PU	23.5	5.7
3% SBE	26.2	5.6
6% SBE	22.6	4.1
9% SBE	15.4	2.7
6% VAE + 8% SF	54.4	9.2
6% VAE + 12% SF	54.3	9.0
6% VAE + 16% SF	65.1	7.71
3% SBR + 8% SF	53.1	7.8
3% SBR + 12% SF	56.4	8.4
3% SBR + 16% SF	64.3	10.4
6% PU + 8% SF	52.3	7.1
6% PU + 12% SF	58.7	8.11
6% PU + 16% SF	58.2	9.16
3% SBE + 8% SF	50.5	7.13
3%SBE + 12%SF	45.2	5.39
3% SBE + 16% SF	32.1	5.86

**Table 10:** Effects of polymers on the mechanical properties of cementitious composites.

Polymers	Polymers (P/C)	Mechanical Properties	Enhancement	Reference
			28 d (Mpa)	
Polyacrylamide	0, 0.2%, 0.5%, 1%, 2%, 3%	Compressive Strength	−9.9	[44]
		flexural strength	0.41	
PSBAMA	0, 0.02%, 0.04%, 0.06%, 0.08%	Compressive Strength	−23	[45]
		flexural strength	2.18	
PSEHAMA	0, 0.02%, 0.04%, 0.06%, 0.08%	Compressive Strength	−31	
		flexural strength	2.6	
VAE	0, 3%, 6%, 9%	Compressive Strength	−4	-
		flexural strength	1	
SBR	0, 3%, 6%, 9%	Compressive Strength	−13.6	-
		flexural strength	−2.2	
PU	0, 3%, 6%, 9%	Compressive Strength	−19.9	-
		flexural strength	−1.6	
SBE	0, 3%, 6%, 9%	Compressive Strength	−30.6	-
		flexural strength	−3.6	

**Table 11:** Interfacial pull-off bond strength.

Coating Materials	Coating Thickness (mm)	Interfacial Pull-Off Bond Strength/MPa		
		28 d	56 d	90 d
blank	/	1.68	1.82	1.85
OCCM	1	1.59	1.85	1.97
	3	1.70	2.15	2.22
	5	1.69	1.97	2.18
6% VAE + 8% SF	1	2.01	2.39	2.66
	3	2.19	2.65	3.18
	5	2.33	2.76	3.01
3% SBR + 8% SF	1	2.10	2.37	2.74
	3	2.59	2.67	2.88
	5	2.36	2.63	2.85
6% PU + 12% SF	1	1.76	1.97	2.35
	3	1.84	2.33	2.97
	5	2.11	2.40	2.76
3% SBE + 8% SF	1	1.76	1.96	2.15
	3	2.41	2.54	2.71
	5	2.27	2.59	2.83

**Table 12:** Water absorption rate of coating materials with different VAE content.

VAE content/%	0	3	6	9
Water absorption rate/%	4.41	2.59	2.53	1.83

**Table 13:** Loss of strength after painting.

Coated Materials	Paint Thickness/mm	Strength Loss Rate/%		
		28 d	56 d	90 d
blank	/	7.21	10.92	15.81
OCCM	1	7.03	9.02	12.19
	2	5.68	7.06	10.73
	3	5.37	7.71	10.45
6% VAE + 8% SF	1	4.15	7.30	9.29
	2	3.43	3.90	6.73
	3	3.49	3.46	6.56
3% SBR + 8% SF	1	4.93	8.52	9.77
	2	4.02	4.99	8.25
	3	3.62	4.86	8.13
6% PU + 12% SF	1	3.21	5.64	9.70
	2	2.18	3.59	7.55
	3	1.97	3.71	6.11
3% SBE + 8% SF	1	5.09	9.57	11.10
	2	4.83	6.86	9.14
	3	4.17	6.64	8.75

## Data Availability

The original contributions presented in this study are included in the article. Further inquiries can be directed to the corresponding author.
